# The mechanism of phonetic information in voice identity discrimination: a comparative study based on sighted and blind people

**DOI:** 10.3389/fpsyg.2024.1352692

**Published:** 2024-05-23

**Authors:** Lili Ming, Libo Geng, Xinyu Zhao, Yichan Wang, Na Hu, Yiming Yang, Xueping Hu

**Affiliations:** ^1^School of Linguistic Sciences and Arts, Jiangsu Normal University, Xuzhou, China; ^2^Key Laboratory of Language and Cognitive Neuroscience of Jiangsu Province, Collaborative Innovation Center for Language Ability, Xuzhou, China; ^3^School of Preschool and Special Education, Kunming University, Yunnan, China; ^4^College of Education, Huaibei Normal University, Huaibei, China; ^5^Anhui Engineering Research Center for Intelligent Computing and Application on Cognitive Behavior (ICACB), Huaibei, China

**Keywords:** voice identity discrimination, language familiarity effect, phonological short-term memory, blind people, sighted people

## Abstract

**Purpose:**

The purpose of this study is to examine whether phonetic information functions and how phonetic information affects voice identity processing in blind people.

**Method:**

To address the first inquiry, 25 normal sighted participants and 30 blind participants discriminated voice identity, when listening forward speech and backward speech from their own native language and another unfamiliar language. To address the second inquiry, combining articulatory suppression paradigm, 26 normal sighted participants and 26 blind participants discriminated voice identity, when listening forward speech from their own native language and another unfamiliar language.

**Results:**

In Experiment 1, not only in the voice identity discrimination task with forward speech, but also in the discrimination task with backward speech, both the sighted and blind groups showed the superiority of the native language. This finding supports the view that backward speech still retains some phonetic information, and indicates that phonetic information can affect voice identity processing in sighted and blind people. In addition, only the superiority of the native language of sighted people was regulated by the speech manner, which is related to articulatory rehearsal. In Experiment 2, only the superiority of the native language of sighted people was regulated by articulatory suppression. This indicates that phonetic information may act in different ways on voice identity processing in sighted and blind people.

**Conclusion:**

The heightened dependence on voice source information in blind people appears not to undermine the function of phonetic information, but it appears to change the functional mechanism of phonetic information. These findings suggest that the present phonetic familiarity model needs to be improved with respect to the mechanism of phonetic information.

## Introduction

Linguistic information (what is being said) and voice identity information (who is speaking) are both important information transmitted by human voices (Kuhl, 2011). The perception of this information is particularly important for blind people. Studies from ordinary people have shown that linguistic information, especially phonetic information, can affect voice identity processing from top to bottom (Scott, 2019). This also can be seen in some studies about phonagnosia and dyslexia. Patients with phonagnosia were not reported to have obvious phonetic processing impairment (Roswandowitz et al., 2014). Patients with dyslexia not only have obvious deficits in phonetic processing (Kita et al., 2013; Cao et al., 2017), but also in native voice identity recognition (Perrachione et al., 2011). Moreover, their performance of phonetic processing represented by phonological short-term memory (pSTM) and phonological awareness (PA) can predict their performance of native voice identity recognition (Perrachione et al., 2011; Perea et al., 2014). These results provide some evidence suggesting that phonetic information may have a top-down impact on voice identity processing.

In addition to the patients with deficits in phonetic processing, the language familiarity effect of voice identity processing (hereinafter referred to as LFE) in ordinary people also supports this viewpoint. LFE means that listeners perform better in native voice identity processing compared to that of nonnative voice identity processing. The superiority of the native language was first proposed by Thompson (1987) and has been widely demonstrated in the field of voice identity recognition (Goggin et al., 1991; Zarate et al., 2015) and voice identity discrimination (Winters et al., 2008; Wester, 2012; Sharma et al., 2020).

The gain effect of the phonetic information of native language on voice identity processing is generally considered the explanation for the LFE. On the one hand, many studies have demonstrated the interpretability of phonetic information. For example, whether it is the distance of the language family between two languages or the time of exposure to a second language, when the phonetic information of nonnative language becomes more familiar for listeners, the superiority of the native language is significantly weakened (Bregman and Creel, 2014; Zarate et al., 2015; de Seyssel et al., 2022). On the other hand, many studies have also shown the nonnecessity of interpretation of semantic information and its interpretation dependence on phonetic information. For example, some studies on 4.5-month-old infants have demonstrated that even if higher-level semantic representations are not established, some developed phonetic skills of native language have already induced the superiority of the native language (May et al., 2011; Fecher et al., 2019). Other adult studies also demonstrated that even in the case of semantic inability to perceive, the difference in phonetic information between native and nonnative language represented in backward speech have also shown the superiority of the native language (Fleming et al., 2014; Hu et al., 2017). Notably, backward speech has been shown to retain some phonetic information, especially for phonemes with time-symmetric properties such as fricatives and nasals (Black, 1973; Ishida, 2021). Therefore, the backward speech of native language can at least be recognized by native listeners as fricatives and nasals. However, for the backward speech of nonnative language, only the part of the phonemes that is the same as these fricative and nasal phonemes of native language can be recognized by native listeners. Thus, there is a difference in the amount of recognizable phonetic information between native and nonnative backward speech, which may be further manifested as the superiority of the native language (Bricker and Pruzansky, 1966). In addition to the case of semantic stripping, studies on semantic retention have also shown the interpretability of semantic information (Goggin et al., 1991; Perrachione et al., 2015; Zarate et al., 2015). However, more importantly, the interpretation of semantic information still needs to be rooted in phonetic information. McLaughlin et al. (2019) used Mandarin-English hybrid speech stimuli for voice identity recognition research. This hybrid speech stimulus were spoken in nonnative language-Mandarin (for example, “陪你晚到了”), with native language-English subtitles that are similar to Mandarin pronunciation (for example, “Pay me one dollar”). Such kind of hybrid stimuli were acoustically unfamiliar but semantically understandable. Their results showed that even after 3 days of voice identity training, the accuracy of voice identity recognition under hybrid condition is similar with unfamiliar language conditions, indicating that semantic representation without familiar phonetic forms does not affect LFE. Therefore, the evidence from both phonetic and semantic perspectives showed that the gain effect of phonetic information is likely to be the cognitive explanation for LFE.

Does the phonetic information of native language always and necessarily have a gain effect on voice identity processing, especially for blind listeners who have the advantage of voice source processing? In addition to phonetic information, voice source information is also crucial for voice identity processing. Voice source information refers to speaker-related vocal fold parameters (such as fundamental frequency) and vocal tract parameters (such as vocal tract length) (Mathias and von Kriegstein, 2014). Previous studies have found that under unfamiliar language conditions where phonetic information is not accessible, blind people performed significantly better in voice identity recognition than normal sighted people (Zhang et al., 2021). It indicates that blind people have an advantage in processing voice identity through voice source information. In addition, under pseudoword conditions where phonetic information is accessible, blind people also performed significantly better than normal sighted people (Föcker et al., 2012, 2015; Hölig et al., 2014a,b). However, in these studies, it has not been further clarified whether the advantage of voice identity processing in blind people comes from the utilization of voice source information or phonetic information. Among this question, it is also not clear whether blind listeners’ voice identity processing uses phonetic information. Notably, under native language conditions where phonetic information is accessible, the performance of native voice identity recognition is significantly correlated with that of nonnative voice identity recognition in blind people (Zhang et al., 2021). This result indicates that even if phonetic information is available, blind people may still rely more on voice source information for voice identity processing. Thus, for blind people, is it still necessary to rely on phonetic information for voice identity processing? In other words, does phonetic information still act on voice identity processing in blind people?

Furthermore, if phonetic information acts on voice identity processing in blind people, does phonetic information act on voice identity processing through pSTM in blind people? Previously, many behavioral studies reported a positive correlation between pSTM and native voice identity recognition performance (Perrachione et al., 2011; Levi, 2014; El Adas and Levi, 2022). Some neuroimaging studies also found that the brain regions related to pSTM, namely the left inferior frontal gyrus and the left precentral gyrus (Fegen et al., 2015; Nishida et al., 2017), have higher accuracy of native voice identity classification than theoretical chance level (Aglieri et al., 2021). Therefore, we have reasons to infer that pSTM may be an important way for phonetic information to act on voice identity processing. Can these results and inferences based on sighted people be analogized to blind people? In other words, is pSTM also an important way for phonetic information to act on voice identity processing in blind people? At present, the issue has not been explained in the phonetic familiarity model that supports the role of phonetic information (Johnson et al., 2011; Fleming et al., 2014).

In summary, based on the LFE phenomenon, we conducted two behavioral experiments on whether phonetic information functions and how acts on voice identity processing in normal-sighted and blind people.

In Experiment 1, we attempted to answer the question of whether phonetic information acts on voice identity processing in sighted and blind people. According to the studies of Fleming et al. (2014) and Hu et al. (2017), we used backward speech to observe whether backward speech bring out LFEs in sighted and blind groups. If the results show that the blind group also exhibits LFE in backward speech, then phonetic information acts on voice identity processing in blind people, which is similar with that of sighted people.

In Experiment 2, we tried to further address how phonetic information acts on voice identity processing in sighted and blind people. The situation is embodied as whether phonetic information can act on voice identity processing through pSTM in sighted and blind people. We used the articulatory suppression paradigm to prevent pSTM (Baddeley, 2003; Kattner and Ellermeier, 2014) and then observed whether the suppression of pSTM led to a decrease in LFEs in the sighted and blind groups. If the results show that after pSTM is suppressed both sighted and blind groups show a decrease in LFEs, then this indicates that phonetic information can act on voice identity processing through pSTM. In addition, the principles and operations of the articulatory suppression paradigm were specifically explained in Experiment 2.

## Experiment 1: does phonetic information affect voice identity processing in sighted and blind people?

### Method

#### Participants

Sample size was determined with power analysis by using GPower 3.1. Assuming α (0.05), statistical test power (1-β, 90%), and effect size (*f* = 0.25), we calculated the sample size required for each group as 23. To improve the statistical testing power, 25 normal sighted participants (5 females, mean age 33 ± 13) and 30 blind participants (5 females, mean age 37 ± 11) were recruited. There was no significant difference in age between the two groups, *t*_(1, 53)_ = −1.374, *p* = 0.175, Cohen’s *d* = 0.372, 95% confidence interval [CI] [−11.18, 2.09], and no significant difference in years of education between the two groups, *t*_(1, 53)_ = 0.974, *p* = 0.335, Cohen’s *d* = 0.264, 95% [CI] [−0.77, 2.22]. All participants were native Chinese speakers, right-handed, without a history of brain disease or neurological illness, with normal bilateral hearing (0.25–4 kHZ, average pure tone hearing threshold ≤25 dB). Experiments 1 and 2 were approved by the local ethics committee.

#### Stimuli

The Chinese and Turkish speech materials were selected from OSCAAR (The Online Speech/Corpora Archive and Analysis Resource).^1^ For each language, five sentences were selected from the corresponding corpus, and each sentence was recorded by 5 male native speakers to avoid influence from paralinguistic information of voices, such as gender.

Then, speech materials were sampled at 16 bit, 22.05 kHZ, time-reversed, and normalized for root-mean-square (RMS) amplitude to 70 dB SPL. The average speech duration of the two languages is as follows: The average speech duration of Chinese is (1,509 ± 19) ms, and while that of Turkish is (1,502 ± 10) ms. Other acoustic characteristics were shown in Table 1.

**Table 1:** Average acoustic characteristics of speech materials.

Speaker	Mandarin			Turkish		
	F0(*M ± SD*)	FD	HNR	F0(*M ± SD*)	FD	HNR
1	147.32 ± 8.39	1.05	7.01	138.01 ± 6.82	1.03	9.90
2	115.32 ± 6.25	1.00	7.61	115.17 ± 4.36	0.98	8.09
3	120.77 ± 4.66	1.00	7.10	137.06 ± 9.48	1.01	9.13
4	142.97 ± 5.65	1.01	10.07	131.88 ± 6.08	0.97	7.39
5	110.43 ± 2.97	0.97	9.13	122.15 ± 1.89	0.95	9.69
*Mean*	127.36 ± 16.71	1.00	8.18	128.85 ± 9.91	0.99	8.84

In addition, we matched the spatial distance of voice identities between two languages to ensure similarity in distribution. The calculation of the spatial distance of voice identities is based on a widely recognized viewpoint, which is that voice identity is a gestalt-like overall representation rather than determined by a specific feature (Fontaine et al., 2017). Moreover, studies have shown that there are three important acoustic parameters for voice identity similarity perception, namely fundamental frequency (F0), resonant dispersion (FD) and harmonic-to-noise ratio (HNR) (Perrachione et al., 2019). Therefore, we considered the specific speaker’s voice identity as a point in the three-dimensional space composed of above three acoustic parameters. The spatial distance of voice identities between two speakers can be calculated by the Euclidean distance between two points in the three-dimensional spaces. Combined with our study, the expression of the spatial distance of voice identities between two speakers is:


$$d=\sqrt[{}]{{\left(F01-F02\right)}^{2}+{\left(FD1-FD2\right)}^{2}+{\left(HNR1-HNR2\right)}^{2}}$$

Note: The acoustic parameters related to the first speaker’s voice identity are F01, FD1, and HNR1, while the acoustic parameters related to the second speaker’s voice identity are F02, FD2, and HNR2.

Since the speech materials of each language were recorded by 5 speakers, the spatial distance of voice identities between two speakers in each language yielded 10 combinations according to pairwise combination. Based on these 10 combinations, we calculated the average spatial distance of voice identities (*M ± SD*) and the confidence interval for each language: the average spatial distance of voice identities between Chinese speakers is 41.74 ± 22.77, and the 95% confidence interval is [29.50, 55.50]. The average spatial distance of voice identities between Turkish speakers is 43.83 ± 21.88, and the 95% confidence interval is [31.24, 56.31]. In view of the comparable average spatial distance and confidence interval of voice identities in the two languages, we considered that voice identities of the two languages have a relatively similar distribution in acoustic parameters.

#### Procedure

The 5 sentences of each language were recorded by 5 male native speakers, so each language had 30 pairs of the same trial (a pair of stimuli from the same speaker) and 30 pairs of different trials (a pair of stimuli from different speakers). Therefore, 120 pairs of forward stimuli and 120 pairs of backward stimuli were obtained. These pairs of stimuli were mixed and divided into 3 blocks.

**Figure 1:**
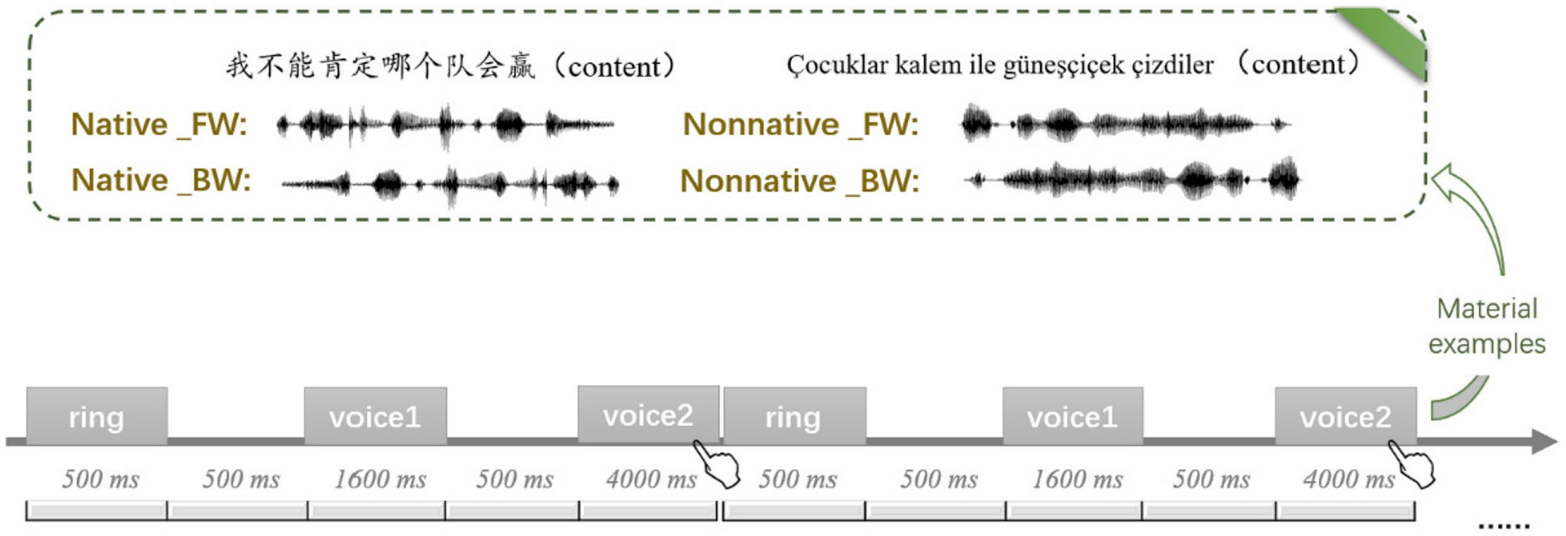
Flow chart of voice identity discrimination task in Experiment 1. Regarding the label of condition, Native and Nonnative represent native language and nonnative language respectively, FW and BW represent forward speech and backward speech, respectively.

The instructions were provided to participants before the experiment. Only after participants correctly understood and passed the practice task (accuracy ≥70%) could they participate in the formal experiment. The process of practice tasks and formal experiments was as follows: Each trial started with a warning ring lasting for 500 ms, followed by a 500 ms blank screen. Afterward, the first speech stimulus was displayed for 1,600 ms, followed by a 500 ms blank screen. Then, the second speech stimulus was displayed for 4,000 ms, during which participants were told to discriminate whether the two speakers were the same or different and to respond by pressing the “1” key for “same trial” with the right index finger and the “3” key for “different trial” with the right middle finger (Figure 1). Each block took 5 min to complete, with a 2-min break between two blocks, so the total task took approximately 20 min.

### Results

After eliminating the data beyond 3 standard deviations, three-factor repeated measurement ANOVA was performed for accuracy and response time. Sometimes, based on the significant interaction of two/three factors, we calculated the LFE value for further comparison to intuitively show how the LFE was affected by other factors (McLennan and Luce, 2005; McLennan and González, 2012). Specifically, the LFE value was obtained by subtracting the accuracy/response time of nonnative language from that of native language.

#### Accuracy

Repeated measurement of 2 (language: native language, nonnative language) × 2 (play manner: forward speech, backward speech) × 2 (group: blind group, sighted group) ANOVA was performed for accuracy. The results showed that the main effect of language was significant, *F*_(1, 53)_ = 353.311, *p* < 0.001, *η*^2^ = 0.870; this manifested in the fact that native language was more accurately discriminated than was nonnative language. The main effect of play manner was significant, *F*_(1, 53)_ = 106.306, *p* < 0.001, *η*^2^ = 0.667; this manifested in the fact that forward speech was more accurately discriminated than was backward speech. The main effect of the group was not significant, *F*_(1, 53)_ = 1.716, *p* = 0.196, *η*^2^ = 0.031.

The interaction effect between language and play manner was significant, *F*_(1, 53)_ = 19.953, *p* < 0.001, *η*^2^ = 0.274. Specifically, the difference between native and nonnative language, namely LFE, was significant in both forward speech and backward speech (*p* < 0.001). However, LFE was significantly larger in forward speech (+0.17) than that in backward speech (+0.12), *t*_(1, 54)_ = 3.987, *p* < 0.001, Cohen’s *d* = 0.538, 95% [CI] [−0.08, −0.03]. The interaction effect between language and group was not significant, *F*_(1, 53)_ = 0.648, *p* = 0.424, *η*^2^ = 0.012. The interaction effect of play manner and group was not significant, *F*_(1, 53)_ = 0.086, *p* = 0.771, *η*^2^ = 0.002.

**Figure 2:**
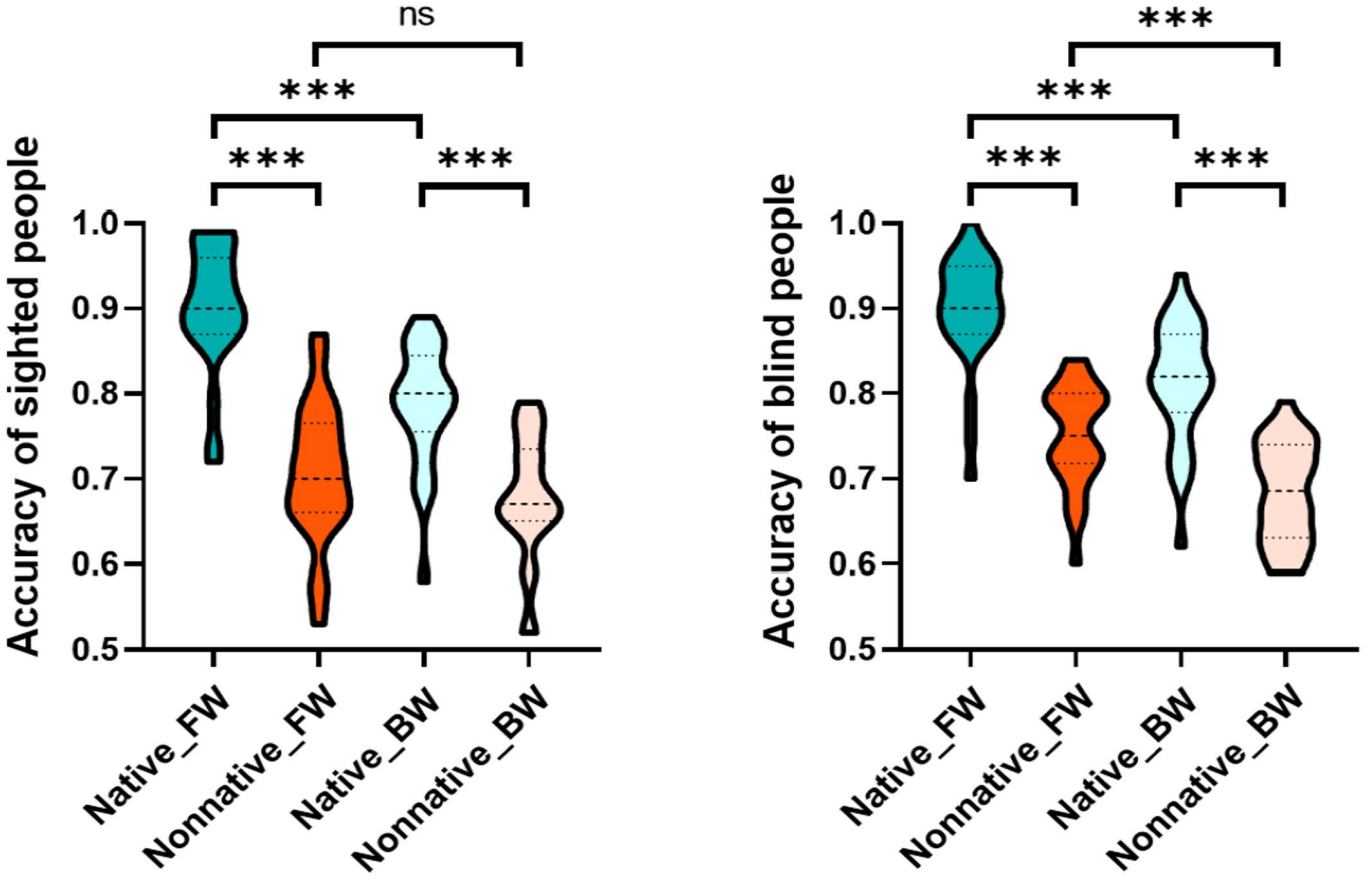
Interaction effect of language and play manner in the sighted and blind groups. The picture on the left shows the interaction effect between language and play manner in the sighted group, and the picture on the right shows the interaction effect between language and play manner in the blind group. Regarding the label of condition, Native and Nonnative represent native language and nonnative language respectively, FW and BW represent forward speech and backward speech, respectively, *p ≤ 0.05, **p ≤ 0.01, ***p ≤ 0.001.

The interaction effect of language, play manner and group was significant, *F*_(1, 53)_ = 7.806, *p* = 0.007, *η*^2^ = 0.128. Then, we reduced the dimension of the group factor, and analyzed the interaction effect of language and play manner for the sighted and blind groups to explore how the LFE of each group was affected by the play manner. After dimensionality reduction, the results showed that the interaction effect between language and play manner was significant in the sighted group, *F*_(1, 24)_ = 31.405, *p* < 0.001, *η*^2^ = 0.567. Specifically, LFE was significant in both forward speech and backward speech (*p* < 0.001). However, LFE was significantly larger in forward speech (+ 0.20) than that in backward speech (+ 0.11), *t*_(1, 24)_ = 5.604, *p* < 0.001, Cohen’s *d* = 1.121, 95% [CI] [−0.12, −0.06]. This phenomenon was because compared with that of forward speech, the accuracy of native language in backward speech was significantly decreased (*p* < 0.001), while the accuracy of nonnative language in backward speech did not significantly change (*p* > 0.05). There was no significant interaction between language and play manner in the blind group, *F*_(1, 29)_ = 1.293, *p* = 0.265, *η*^2^ = 0.043 (Table 2 and Figure 2).

**Table 2:** Average accuracy and response time of voice identity discrimination (*M ± SD*) in Experiment 1.

Group	Language	Forward speech		Backward speech	
		ACC	RT	ACC	RT
Sighted	Native	0.90 ± 0.07	1,138 ± 328	0.79 ± 0.07	1,130 ± 303
	Nonnative	0.71 ± 0.08	1,143 ± 286	0.68 ± 0.07	1,225 ± 323
Blind	Native	0.90 ± 0.06	1,048 ± 291	0.81 ± 0.07	1,084 ± 292
	Nonnative	0.75 ± 0.06	1,119 ± 299	0.68 ± 0.06	1,192 ± 333

#### Response time

Repeated measurement of 2 (language: native language, nonnative language) × 2 (play manner: forward speech, backward speech) × 2 (group: blind group, sighted group) ANOVA was performed for the response time. The results showed that the main effect of language was significant, *F*_(1, 53)_ = 67.696, *p* < 0.001, *η*^2^ = 0.561, which showed that native language was discriminated faster than nonnative language. The main effect of play manner was significant, *F*_(1, 53)_ = 19.432, *p* < 0.001, *η*^2^ = 0.268; this manifested in the fact that forward speech was discriminated faster than backward speech. The main effect of group was not significant, *F*_(1, 53)_ = 0.351, *p* = 0.556, *η*^2^ = 0.007.

The interaction effect between language and play manner was significant, *F*_(1, 53)_ = 8.726, *p* = 0.005, *η*^2^ = 1.141. Specifically, LFE was significant in both the forward speech (*p* = 0.018) and backward speech (*p* < 0.001). However, LFE was significantly smaller in forward speech (−102 ms) than that in backward speech (−41 ms), *t*_(1, 54)_ = −2.841, *p* = 0.006, Cohen’s *d* = 0.383, 95% [CI] [−104.42, −18.02]. The interaction effect between language and group was significant, *F*_(1, 53)_ = 5.594, *p* = 0.022, *η*^2^ = 0.095. LFE was significant in both the blind group and the sighted group (*p* < 0.001). However, the LFE (−90 ms) of the blind group was significantly smaller than that of the sighted group (−50 ms), *t*_(1, 53)_ = 2.365, *p* = 0.022, Cohen’s *d* = 0.641, 95% [CI] [12.16, 147.80]. The interaction effect between play manner and group was not significant, *F*_(1, 53)_ = 0.741, *p* = 0.393, *η*^2^ = 0.014. The interaction effect of language, play manner and group was not significant, *F*_(1, 53)_ = 1.505, *p* = 0.225, *η*^2^ = 0.028 (Table 2).

### Discussion

The results of Experiment 1 showed that backward speech with some phonetic information retained can make both sighted and blind people exhibit LFE in terms of the accuracy index. The result is similar with those obtained by Fleming et al. (2014) and Hu et al. (2017), and suggests that phonetic information also plays an important role in voice identity processing for blind people, similar with that in sighted people.

At the same time, the accuracy index also showed that compared with forward speech, backward speech significantly reduced the LFE of sighted people, especially with a negative impact on their native language. However, it did not significantly influence the LFE of blind people. In fact, it is unclear whether this behavior pattern of backward speech originates from phonological factors or semantic factors, as both information are seriously impaired by reversing. Based on this result, we decided to further explore the potential reasons for the patterns of backward speech from the phonological perspective. The reasons are as follows.

Firstly, backward speech requires reversing the pronunciation order of phonemes, which is essentially due to the impaired phonological information, resulting in the damage of semantic information. Therefore, we attempted to explore the reasons from the phonological perspective at a deeper level.

Secondly, backward speech has been recognized by many researchers as lacking reproducibility. Functional magnetic resonance imaging studies also showed that compared with real words and pseudowords, backward speech lacks integration between parietal-somatosensory-motor networks and functional connectivity between right cerebellum-motor regions. It has been demonstrated from a neural perspective that backward speech lacks motor reproducibility/rehearsal (Londei et al., 2010). Moreover, in the working memory model, the rehearsal strategy is related to the maintenance of phonological memory, while attentional refresh is related to the maintenance of semantic memory (Haarmann and Usher, 2001; Nishiyama, 2014, 2018; Loaiza and Camos, 2018). Then it is reasonable to believe that the behavior pattern found in backward speech are likely more relevant to phonological memory than semantic memory.

Therefore, based on the results that backward speech only had a negative impact on the accuracy of native voice identity discrimination for sighted people rather than blind people, we decided to explore whether pSTM plays a different role in voice identity processing for different group in Experiment 2. It was proposed that although phonetic information can act on voice identity processing in sighted and blind people, it can act on voice identity processing through pSTM in sighted people only rather than blind people.

## Experiment 2: does phonetic information act on voice identity processing through pSTM in sighted and blind people?

In experiment 2, we tried to further address whether phonetic information can act on voice identity processing through pSTM in sighted and blind people.

To investigate the role of pSTM in the voice identity processing, we mainly used the articulatory suppression paradigm. The working memory model proposes that as a component of the phonological loop, subvocal rehearsal can be used to offset the effects of memory decay (Baddeley et al., 1975). If irrelevant articulations are concurrently uttered during subvocal rehearsal, the process of rehearsal will be prevented (i.e., articulatory suppression). Hence, phonological effects will be abolished (Murray, 1968; Baddeley et al., 1975) and semantic effects are preserved under articulatory suppression (Saint-Aubin and Poirier, 1999; Haarmann and Usher, 2001; Nishiyama, 2014, 2018). Based on the sufficient empirical research, we believed that it is reasonable to use the articulatory suppression paradigm to explore the role of pSTM. It was expected that manipulation of pSTM would regulate the LFE of sighted people rather than blind people.

In addition to the experimental manipulation of pSTM activity, we also conducted behavioral assessments of pSTM ability (i.e., nonword repetition task). It was expected that pSTM scores were positively correlated with native voice identity discrimination performance under control conditions, which may only occur in the sighted group rather than blind group.

In sum, the two methods had the effect of mutual verification and jointly demonstrate the main purpose of this experiment.

### Method

#### Participants

The selection criteria for sample size were the same as in Experiment 1. Thus, we calculated the sample size required for each group as 23. To improve the statistical testing power, 26 normal sighted participants (3 females, mean age 32 ± 14) and 26 blind participants (5 females, mean age 36 ± 11) were recruited. There was no significant difference in age between the two groups, *t*_(1, 50)_ = 1.056, *p* = 0.296, Cohen’s *d* = 0.293, 95% [CI] [−10.71, 3.33], and no significant difference in years of education between the two groups, *t*_(1, 50)_ = 0.858, *p* = 0.395, Cohen’s *d* = 0.238, 95% [CI] [−0.88, 2.18]. All participants were native Chinese speakers, right-handed, without a history of brain disease or neurological illness, with normal bilateral hearing (0.25–4 kHz, average pure tone hearing threshold ≤25 dB).

#### Stimuli

The selection and combination of stimuli were the same as in Experiment 1.

#### Procedure

##### Voice identity discrimination

The experiment consisted of 6 blocks, which were 3 articulatory control blocks and 3 articulatory suppression blocks. Since each block contained all mixed pairs of stimuli (10 pairs of the same trial and 10 pairs of different trials for each language), each block included a total of 40 pairs of stimuli.

**Figure 3:**
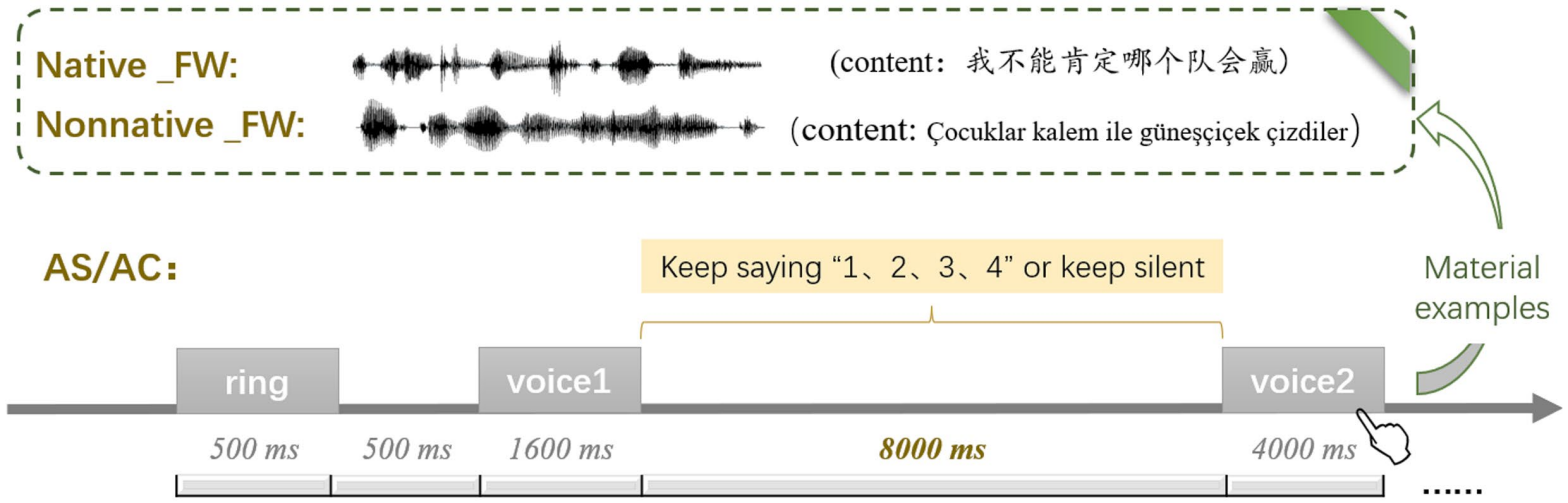
Flow chart of voice identity discrimination task in Experiment 2. Regarding the label of condition, Native and Nonnative represent native language and nonnative language respectively, FW and BW represent forward speech and backward speech respectively, AS and AC represent articulatory suppression and articulatory control, respectively.

The instructions were provided to participants before the experiment. Only after participants correctly understood and passed the practice task (accuracy ≥70%) could they participate in the formal experiment. The process for practice tasks and the formal experiments was as follows: Each trial started with a warning ring lasting for 500 ms, followed by a 500 ms blank screen, and then a 1,600 ms first speech stimulus. Afterward, a blank screen of 8,000 ms was presented. The difference between articulatory control blocks and articulatory suppression blocks was as follows: If the block was an articulatory control block, the participants needed only to wait quietly. If the block was an articulatory suppression block, then the participants needed to keep saying the numbers “1, 2, 3, 4,” and the rhythm of saying the numbers was controlled at approximately 1 number/s. After the 8,000 ms blank screen, the second speech stimulus was displayed for 4,000 ms, during which participants were told to discriminate whether the two speakers were the same or different and to respond by pressing the “1” key for “same trial” with the right index finger, and the “3” key for “different trial” with the right middle finger (Figure 3). Each block took 8 min to complete, with a 2-min break between two blocks, so the total task took approximately 60 min.

##### Phonological short-term memory

Phonological short-term memory is defined as the maintenance of language sounds (Perrachione et al., 2017). The measurement tasks, testing process and scoring criteria referred to existing Chinese related studies (Jiang, 2017). The measurement used a nonword repetition task, where nonwords were meaningless syllable combinations composed of morphemes present in Chinese, such as “ang1 hen4 re4 gei3 cang2.” During the test, the participants were required to repeat a string of syllable combinations as much as possible. The length of the syllable combinations gradually increased from two syllables to ten syllables. In terms of score, correctly repeating a syllable counted as 1 point, while errors counted as 0 points. Then through dividing the number of syllables repeated correctly by the total number of syllables to obtain the percentage of the syllable output which was recorded as their pSTM score.

##### Phonological awareness

Phonological awareness (PA) is defined as the awareness of the structure of language sounds (Wagner and Torgesen, 1987). The measurement tasks, testing process and scoring criteria referred to existing Chinese related studies (Liu et al., 2006; Chen, 2018). The measurement of Chinese PA was divided into three parts: syllable awareness, initials, finals and tone awareness, and phonemic awareness.

The syllable awareness test used a syllable recognition task, which selected one item from two candidate options that has the same syllable as the given target item. For example, according to the target item “shan1 yang2,” participants needed to select one of the two options “da4 shan1” and “qing1 cao3” that had the same syllable as the target, where “da4 shan1” was the correct answer.

The initials, finals and tone awareness used the recognition tasks of initials, finals and tones. Taking the recognition task of initials as an example, participants needed to select one of the two options with the same initials as the given target item. For example, according to the target item “chong2,” participants needed to select one of the two options “shen2” and “chuan2” that had the same initial consonant as the target, where “chuan2” was the correct answer.

Phonemic awareness used a phoneme deletion task, in which the deleted phoneme may be the item at the beginning, end or middle of the syllable. The participants needed to speak the remaining item of the syllable after part of the phonemes had been deleted. For example, according to the target item “jiao1,” after the phoneme “o” was deleted by researchers, the participants answered “jia1” or “jia3,” regardless of whether the tone changed or not.

After the above tests were completed, the total score obtained in all tasks was recorded as their PA score. Each task included 15 questions, with 1 point for each correctly answered question and 0 points for each incorrectly answered question.

### Results

#### Accuracy

Repeated measurement of 2 (language: native language, nonnative language) × 2 (articulatory rehearsal: articulatory control, articulatory suppression) × 2 (group: blind group, sighted group) ANOVA was performed for accuracy. The results showed that the main effect of language was significant, *F*_(1, 50)_ = 375.291, *p* < 0.001, *η*^2^ = 0.882; this manifested in the fact that native language was more accurately discriminated than was nonnative language. The main effect of articulatory rehearsal was significant, *F*_(1, 50)_ = 46.621, *p* < 0.001, *η*^2^ = 0.483; this manifested in the fact that articulatory control was more accurately discriminated than articulatory suppression. The main effect of group was not significant, *F*_(1, 50)_ = 0.064, *p* = 0.801, *η*^2^ = 0.001.

The interaction effects of language and articulatory rehearsal, language and group, and articulatory rehearsal and group were not significant, *F*_(1, 50)_ = 0.312, *p* = 0.579, *η*^2^ = 0.006; *F*_(1, 50)_ = 0.374, *p* = 0.544, *η*^2^ = 0.007; *F*_(1, 50)_ = 2.252, *p* = 0.140, *η*^2^ = 0.043. The interaction effect of language, articulatory rehearsal and group was also not significant, *F*_(1, 50)_ = 0.186, *p* = 0.668, *η*^2^ = 0.004 (Table 3).

**Table 3:** Average accuracy and response time of voice identity discrimination (*M ± SD*) in Experiment 2.

Group	Language	Articulatory control		Articulatory suppression	
		ACC	RT	ACC	RT
Sighted	Native	0.82 ± 0.08	1,369 ± 343	0.76 ± 0.08	1,436 ± 343
	Nonnative	0.63 ± 0.07	1,450 ± 319	0.57 ± 0.06	1,446 ± 306
Blind	Native	0.81 ± 0.08	1,248 ± 251	0.77 ± 0.07	1,294 ± 308
	Nonnative	0.63 ± 0.08	1,395 ± 285	0.6 ± 0.06	1,437 ± 309

#### Response time

Repeated measurement of 2 (language: native language, nonnative language) × 2 (articulatory rehearsal: articulatory control, articulatory suppression) × 2 (group: blind group, sighted group) ANOVA was performed for the response time. The results showed that the main effect of language was significant, *F*_(1, 50)_ = 27.263, *p* < 0.001, *η*^2^ = 0.353, which showed that native language was discriminated faster than was nonnative language. The main effect of articulatory rehearsal was significant, *F*_(1, 50)_ = 4.048, *p* = 0.050, *η*^2^ = 0.075. The main effect of group was not significant, *F*_(1, 50)_ = 0.991, *p* = 0.324, *η*^2^ = 0.019.

The interaction effect between language and articulatory rehearsal was significant, *F*_(1, 50)_ = 5.428, *p* = 0.024, *η*^2^ = 0.098. Specifically, LFE was significant in both articulatory control (*p* < 0.001) and articulatory suppression (*p* = 0.002). However, the size of LFE was significantly larger in articulatory control (−114 ms) than that in articulatory suppression (−76 ms), *t*_(1, 51)_ = −2.254, *p* = 0.028, Cohen’s *d* = 0.313, 95% [CI] [−70.57, −4.09]. The interaction effect between language and group was significant, *F*_(1, 50)_ = 7.543, *p* = 0.008, *η*^2^ = 0.131. A *post hoc* test indicated that LFE was significant in the blind group (*p* < 0.001) but not in the sighted group (*p* > 0.05). The interaction effect between articulatory rehearsal and group was not significant, *F*_(1, 50)_ = 0.105, *p* = 0.748, *η*^2^ = 0.002.

**Figure 4:**
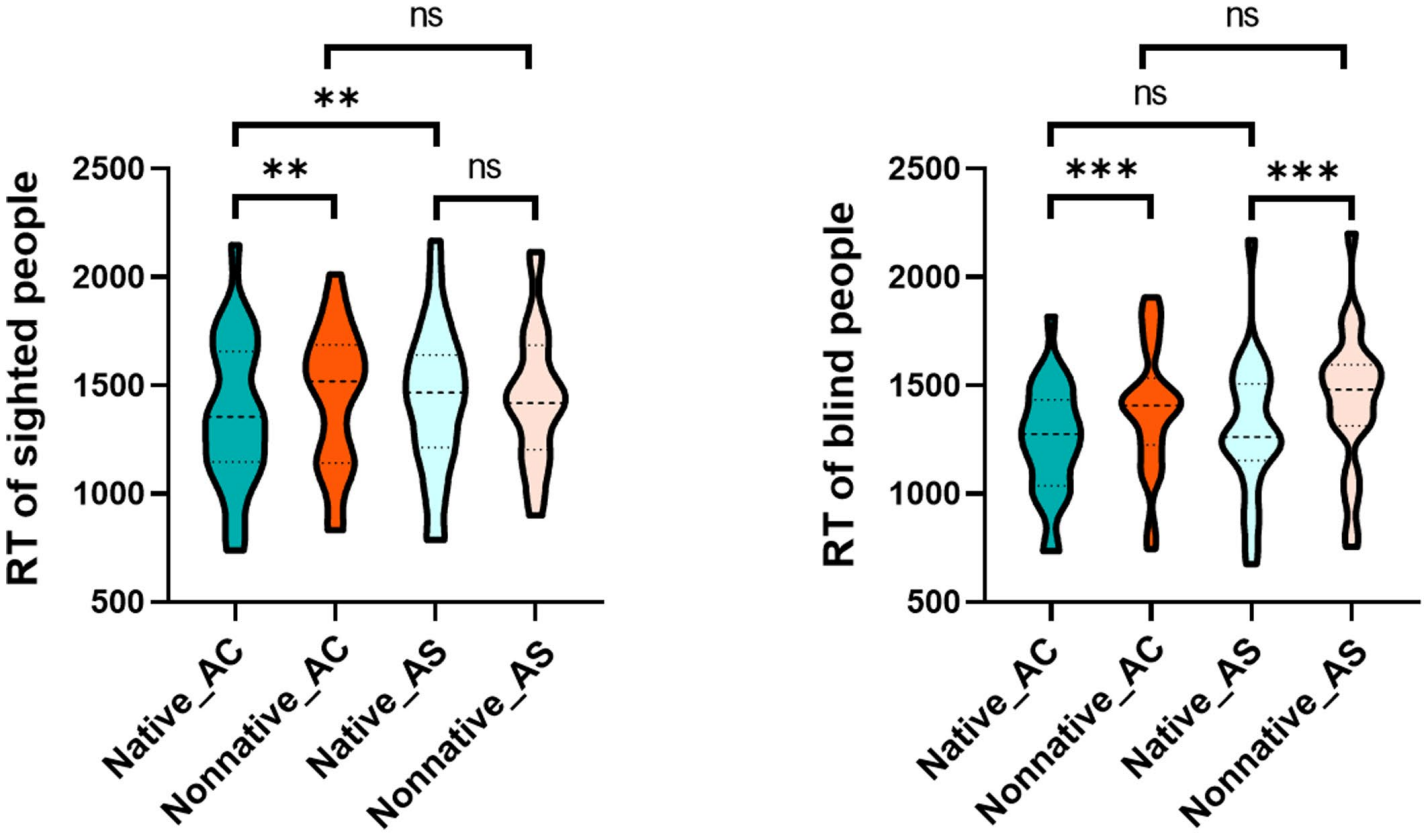
Interaction effect of language and articulatory rehearsal in the sighted and blind groups. The picture on the left shows the interaction effect between language and articulatory rehearsal in the sighted group, and the picture on the right shows the interaction effect between language and articulatory rehearsal in the blind group. Regarding the label of condition, Native and Nonnative represent native language and nonnative language respectively, and AS and AC represent articulatory suppression and articulatory control, respectively, *p ≤ 0.05, **p ≤ 0.01, ***p ≤ 0.001.

The interaction effect of language, articulatory rehearsal and group was significant, *F*_(1, 50)_ = 4.468, *p* = 0.040, *η*^2^ = 0.082. According to the purpose, we reduced the dimension of the group factor, and then analyzed the interaction effect of language and articulatory rehearsal for the sighted and blind groups to explore how the LFE of each group was affected by pSTM. After dimensionality reduction, the results showed that the interaction effect between language and articulatory rehearsal was significant in the sighted group, *F*_(1, 25)_ = 11.918, *p* = 0.002, *η*^2^ = 0.323. A *post hoc* test indicated that LFE was significant in articulatory control (*p* = 0.008) rather than that in articulatory suppression (*p* > 0.05). This phenomenon was because native voice identity discrimination was significantly slower after articulatory suppression (*p* = 0.004), and nonnative voice identity discrimination did not change significantly after articulatory suppression (*p* > 0.05). In addition, the interaction effect between language and articulatory rehearsal was not significant in the blind group, *F*_(1, 25)_ = 0.563, *p* = 0.460, *η*^2^ = 0.022 (Table 3 and Figure 4).

#### Correlation analysis

The performance of native voice identity discrimination in the sighted and blind groups was not the same as that regulated by pSTM, probably due to the different information dependent on native voice identity discrimination. To verify this inference, we conducted Pearson correlation analysis between the accuracy of native voice identity discrimination and pSTM scores. However, considering that native voice identity discrimination may also use vocal source information and other phonological skills such as PA, we combined these four factors to perform Pearson correlation analysis. Here, the ability of vocal source analysis was embodied in the accuracy of nonnative voice identity discrimination, as the strategy of vocal source analysis almost can fully interpretable it. If the results showed that native voice identity discrimination performance was significantly correlated with the other three factors, then these factors are likely to be involved in the identification process.

Before the correlation analysis, we confirmed that there was no significant difference in PA score and pSTM score between the sighted and blind groups, *t*_(1, 50)_ = −0.276, *p* = 0.783, Cohen’s *d* = 0.077, 95% [CI] [−5.40, 4.10]; *t*_(1, 50)_ = 1.570, *p* = 0.123, Cohen’s *d* = 0.435, 95% [CI] [−0.01, 0.08]. It excluded the possibility that the sighted or blind group may rely more on certain phonological skills to discriminate native voice identity due to better phonological skills.

The correlation analysis of the sighted group showed that under the condition of articulatory control, that is, when pSTM was maintained, the accuracy of native voice identity discrimination was significantly correlated with that of nonnative voice identity discrimination (*r* = 0.416, *p* = 0.035) and pSTM (*r* = 0.411, *p* = 0.037). Under the condition of articulatory suppression, that is, when the pSTM was not maintained, the accuracy of native voice identity discrimination was significantly correlated with PA (*r* = 0.572, *p* = 0.002) and pSTM (*r* = 0.551, *p* = 0.004) (Table 4).

**Table 4:** Correlation analysis of the sighted group.

	1. Native_AC	2. Nonnative_AC	3. Native_AS	4. Nonnative_AS	5. PA	6. pSTM
1. Native_AC	–					
2. Nonnative_AC	0.416*	–				
3. Native_AS	0.648**	0.378	–			
4. Nonnative_AS	0.259	0.435*	0.365	–		
5. PA	0.114	0.199	0.572**	0.372	–	
6. pSTM	0.411*	0.304	0.551**	0.004	0.479*	–

Regarding the label of condition, Native and Nonnative represent native language and nonnative language, respectively, AS and AC represent articulatory suppression and articulatory control, respectively. *p ≤ 0.05, **p ≤ 0.01, ***p ≤ 0.001 (significant correlations with purpose is bold).

The correlation analysis of the blind group showed that under the condition of articulatory control, that is, when the pSTM was maintained, the accuracy of native voice identity discrimination was significantly correlated with that of nonnative voice identity discrimination (*r* = 0.709, *p* < 0.001) and PA (*r* = 0.539, *p* = 0.005). Under the condition of articulatory suppression, that is, when the pSTM was not maintained, the accuracy of native voice identity discrimination was significantly correlated with PA (*r* = 0.518, *p* = 0.007) (Table 5).

**Table 5:** Correlation analysis of the blind group.

	1. Native_AC	2. Nonnative_AC	3. Native_AS	4. Nonnative_AS	5. PA	6. pSTM
1. Native_AC	–					
2. Nonnative_AC	0.709***	–				
3. Native_AS	0.760**	0.461*	–			
4. Nonnative_AS	0.460*	0.578**	0.226	–		
5. PA	0.539**	0.312	0.518**	0.322	–	
6. pSTM	0.317	0.223	0.282	0.206	0.527**	–

Regarding the label of condition, Native and Nonnative represent native language and nonnative language respectively, AS and AC represent articulatory suppression and articulatory control, respectively. *p ≤ 0.05, **p ≤ 0.01, ***p ≤ 0.001 (significant correlations with purpose is bold).

## Discussion

The results of Experiment 2 showed that for sighted people, articulatory suppression significantly reduced LFE compared to that of articulatory control in terms of the response time index. This phenomenon was mainly due to a significant increase in the response time of native voice identity discrimination relative to nonnative voice identity discrimination. Therefore, for sighted people, native and nonnative voice identity processing may use different strategies. For example, nonnative voice identity processing mainly depends on voice source memory, while native voice identity processing requires not only voice source memory but also pSTM. This inference was further verified in the correlation analysis; that is, the performance of native voice identity discrimination under the condition of articulatory control was not only correlated with the performance of nonnative voice identity discrimination but also correlated with pSTM.

For blind people, in the accuracy and response time indicators, articulatory suppression had no significant impact on LFE compared to articulatory control. It indicates that their native and nonnative voice identity processing may use similar strategies. For example, native voice identity processing relies on voice source memory rather than pSTM, similar with nonnative voice. The results of correlation analysis also supported the inference; that is, their performance of native voice identity discrimination under the condition of articulatory control was correlated with nonnative voice identity discrimination, but not correlated with pSTM. In addition, their performance of native voice identity discrimination under the condition of articulatory control was significantly correlated with PA, indicating that their native voice identity processing may also rely on PA.

In summary, some our evidence showed that pSTM represented by articulatory rehearsal can regulate the native voice identity discrimination of sighted people rather than blind people. Moreover, pSTM was significantly correlated with native voice identity discrimination in sighted people rather than blind people. Above evidence suggests that phonetic information can act on voice identity processing through pSTM in sighted people rather than blind people.

## General discussion

Previous studies have shown that phonetic information can promote voice identity processing. Does this conclusion apply to blind people who rely more on voice source information for voice identity processing? Then if phonetic information works, how does phonetic information act on voice identity processing in blind people? Therefore, through two behavioral experiments, we demonstrated in this article that blind people’s high dependence on voice source information does not lead to the elimination of the role of phonetic information, but is likely to change the functional mechanism of phonetic information.

### Phonetic information acts on voice identity processing in sighted and blind people

For sighted people, some phonetic information retained in backward speech can make them exhibit LFE. It is consistent with the results of Fleming et al. (2014) and Hu et al. (2017), indicating that phonetic information can act on voice identity processing in sighted people. We extended this conclusion to the blind people; that is, we demonstrated for the first time that phonetic information can also act on voice identity processing in blind people.

Combining the results of sighted and blind people, we see that phonetic information is always required for voice identity processing, regardless of whether the individual is highly dependent on voice source information. This phenomenon once again emphasizes the automatic processing of phonetic information. In these voice identity processing tasks, phonetic information has not been required to actively paid attention, but it still promotes voice identity processing (Bregman and Creel, 2014; Zarate et al., 2015; de Seyssel et al., 2022). Moreover, the automatic processing of phonetic information is likely to be a compensation strategy for the relative limitation of identity information transmitted by nonspeech vocalization. For example, Lavan et al. (2019) believed that some acoustic cues such as F0, which are important for voice identity processing, are retained in natural speech vocalization but absent in nonspeech vocalization such as laughter and sigh. Thus, nonspeech vocalization can be combined with more abundant speech information (including phonetic information) to enhance voice identity processing. As shown in the study by Zarate et al. (2015), speech vocalization induced higher accuracy of voice identity recognition than nonspeech vocalization.

### Phonetic information acts on voice identity processing via pSTM in sighted rather than blind people

The results of Experiment 1 showed that phonetic information can act on voice identity processing in sighted and blind people. At the same time, it also inspired us that phonetic information may act on voice identity processing in sighted and blind people through different mechanisms.

In Experiment 2, the manipulation of pSTM regulated the LFE of sighted people, indicating that phonetic information can affect their voice identity processing through pSTM. This finding is not surprising, because some previous behavioral studies have found a significant correlation between pSTM and native voice identity recognition performance (Perrachione et al., 2011; Levi, 2014; El Adas and Levi, 2022). On this basis, small contribution of our research lies in the following: (1) The role of pSTM on native voice identity processing is directly and again verified through the manipulation of articulatory rehearsal. (2) The role of pSTM on native voice identity processing is not only in the field of voice identity recognition (Perrachione et al., 2011; Levi, 2014; Aglieri et al., 2021; El Adas and Levi, 2022) but also in the field of voice identity discrimination.

An important finding of this research is that for blind people, the manipulation of pSTM did not regulate their LFE, indicating that phonetic information does not affect their voice identity processing by pSTM. Whether pSTM was suppressed or not, it had a similar influence pattern on native and nonnative voice identity discrimination in blind people. In addition to the similarity, there was a high correlation between native and nonnative voice identity discrimination (when pSTM was not suppressed) in blind people, which was even higher than that found in sighted people. This correlation finding is also consistent with another study on blind people (Zhang et al., 2021). Thus, we have reasons to believe that for blind people, native voice identity processing may use a strategy similar with nonnative voice identity processing, such as voice source memory.

In general, the native voice identity processing mechanism of blind people is different from that of sighted people. The evidence and potential reasons are reflected in the following two points.

On the one hand, unlike sighted listeners, blind listeners’ native voice identity processing may mainly use a strategy similar with nonnative voice identity, such as voice source memory. We argue that this phenomenon is probably related to the difference in the major modality for identity processing between the two groups. For sighted listeners, identity processing is more stable and accurate in the visual modality than that in the auditory modality (Stevenage et al., 2013; Lavan et al., 2022). Therefore, to compensate for the relative disadvantage of identity processing in the auditory modality, we need more support from speech information. In this study, sighted listeners relied on pSTM to process native voice identity. However, blind people who lack visual experience determine who the speaker is based on auditory voice to a large extent. This long-term experience in auditory modality has enabled them to develop better voice source analysis/memory ability (Zhang et al., 2021). Therefore, blind people may need less support from speech information. In this study, blind people were unlikely to rely on pSTM to process native voice identity.

On the other hand, unlike sighted listeners, blind listeners’ native voice identity processing may also rely on PA. In Experiment 1, we found that native voice identity discrimination benefits from the availability of phonetic information in blind people. In Experiment 2, there was a moderate correlation between native voice identity discrimination and PA in blind people. The correlation between native voice identity processing and PA has been found in patients with dyslexia (Perrachione et al., 2011), but not in normal-sighted adults (in this study, we refer to the results under the conditions of articulatory control) and school-age children (Levi, 2014). Then, combined with relevant results of pSTM, a question worth considering is why PA rather than pSTM is correlated with native voice identity processing in blind people. Both PA and pSTM are correlated with native voice identity processing in patients with dyslexia, while pSTM rather than PA is correlated with native voice identity processing in sighted people.

Regarding the above issues, we argue that the role of PA and pSTM may not be the same in native voice identity processing. Previously, the relative separation between PA and pSTM has been directly demonstrated in the field of phonological processing (Majerus et al., 2003; Channell et al., 2013), so it is likely to also be reflected in the field of voice identity processing. Specifically, both voice identity recognition and voice identity discrimination require memory retrieval of voice instances to make judgments, so memory-related roles such as pSTM may be more prominent (than PA). Correspondingly, the correlation between pSTM rather than PA and native voice identity processing was reported in both normal-sighted adults (in this study, we refer to the results under the conditions of articulatory control) and school-age children (Levi, 2014). When the role of pSTM is not exerted or not fully exerted, implicit PA is required to compensate for this deficiency. For example, in this study blind people who rely more on voice source memory do not need to use pSTM, and they showed the correlation between PA and native voice identity processing. In existing studies, patients with dyslexia who have pSTM impairment cannot rely completely on pSTM, and they showed that pSTM and PA are correlated with native voice identity processing (Perrachione et al., 2011). More intuitively, we found that some our results are similar with the conclusion for patients with dyslexia; that is, when pSTM is suppressed, pSTM and PA are significantly correlated with sighted people’s native voice identity processing. Therefore, the cross-group discussions on patients with dyslexia, blind people and sighted people support the hypothesis we proposed above to a large extent.

Finally, in terms of the respective role of PA, pSTM and vocal source information in voice identity discrimination, we made a tentative summary or discussion. In essence, vocal source information is the necessary information for voice identity discrimination, while phonological information is optional and beneficial information.

The vocal source information may be stored as sensory memory traces, and has been shown to involve brain regions responsible for acoustic feature analysis, namely the posterior temporal sulcus/gyrus, temporal plane, and anterolateral temporal gyrus (Maguinness et al., 2018).

Phonological information may be used through strategies such as pSTM or PA to prompt voice identity discrimination. On the one hand, pSTM allows as much phonological content as possible to be repeated and retained in the brain. Longer and more abundant phonological strings contain more details of variable voice, have been proven to improve voice identity recognition performance (Bricker and Pruzansky, 1966). On the other hand, once phonological memory process is prevented or pSTM ability is impaired, other phonological skills, such as PA, may be recruited. PA is the ability to perceive and manipulate the phonemes that constitute words. When the voice details cannot be obtained through sufficient phonological content from memory storage, it is likely to use or combine with the strategy of PA to extract fine phoneme information in order to obtain certain voice details. However, above statements were inferences based on the results, and it was suggested that more direct methods can be used in the future.

### Supplement to phonetic familiarity model

The present LFE model, that is, the phonetic familiarity model (Perrachione, 2018), does not explain whether the role of phonetic information disappears with the processing advantages and high dependence of voice source information in some groups. In this study, we found that the role of phonetic information does not disappear, but the mechanism of phonetic information is likely to change with the high dependence of voice source memory in blind people.

**Figure 5:**
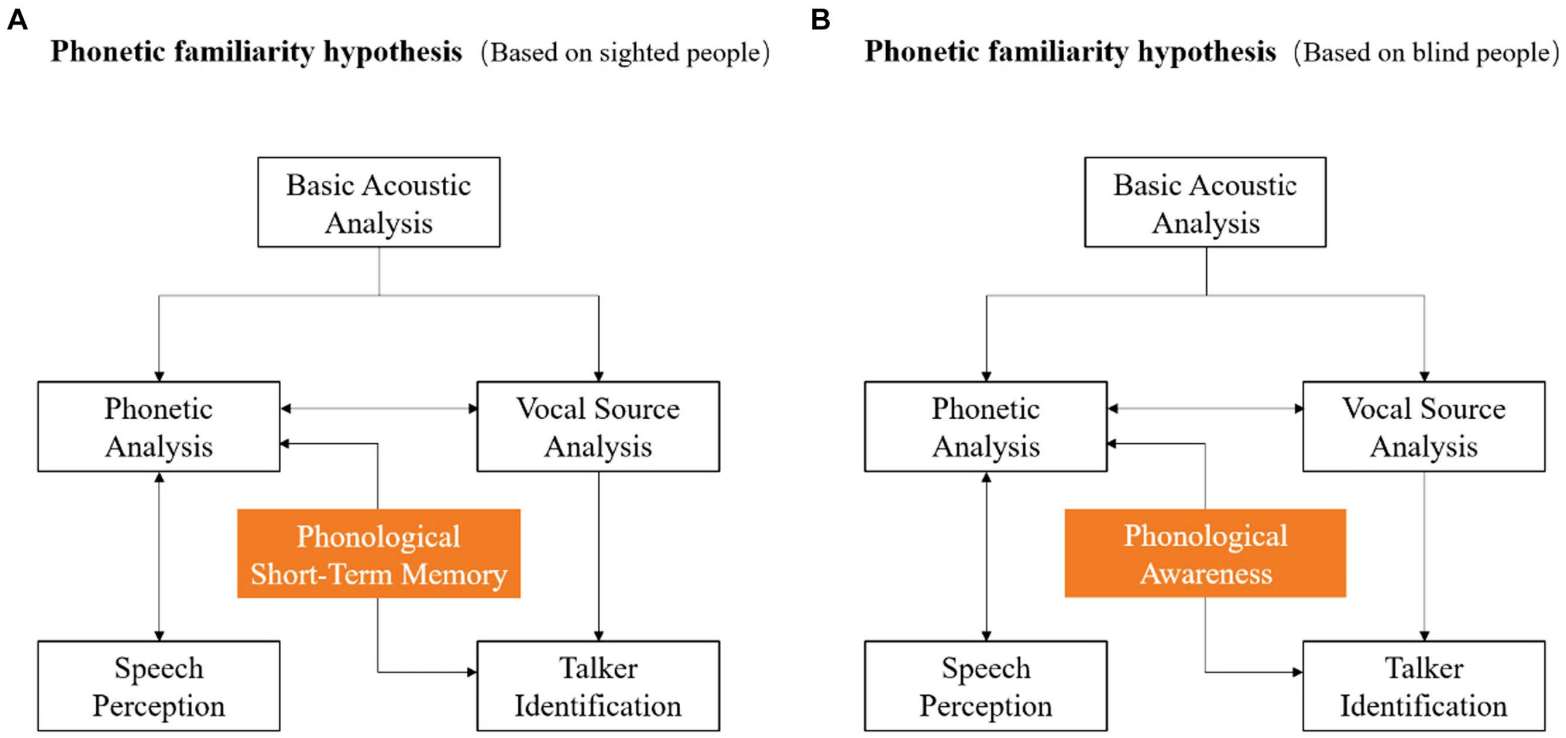
Phonetic familiarity model based on sighted and blind people. **(A)** shows the model based on sighted people, and **(B)** shows the model based on blind people.

According to the findings, this study further improves the specific mechanism of phonetic information in the phonetic familiarity model. Specifically, based on the experimental results of sighted people, the concept of pSTM is added to emphasize its important role. In contrast, based on the experimental results of blind people, the concept of PA is added to emphasize its potential role (Figure 5). In the future, the combination of cognitive training such as pSTM and PA in clinical practice can be considered. It is expected to provide some reference for the rehabilitation treatment of groups with voice identity perception disorders.

## Conclusion

In this study, we found that the role of phonetic information on voice identity processing exists not only in sighted people but also in blind people. However, the specific mechanism of phonetic information is not the same in the two groups. These findings not only support the current phonetic familiarity model, but also suggest that the phonetic familiarity model needs to be further improved relative to the specific mechanism of phonetic information.

Finally, there are still some deficiencies in this study. For example, we explored only the differences in the mechanism of LFE between the two groups from the perspective of pSTM. In the future, we can try to explore the differences between two groups from the perspective of semantic short-term memory. In addition, PA has not been directly demonstrated as an explanatory mechanism for LFE in blind people, which is still worthy of further exploration.

## Data availability statement

The raw data supporting the conclusions of this article will be made available by the authors, without undue reservation.

## Ethics statement

The studies involving humans were approved by Ethical Review Committee of experimental animals of Jiangsu Normal University. The studies were conducted in accordance with the local legislation and institutional requirements. The participants provided their written informed consent to participate in this study. Written informed consent was obtained from the individual(s) for the publication of any potentially identifiable images or data included in this article.

## Author contributions

LM: Conceptualization, Writing – original draft, Data curation, Investigation, Methodology, Validation, Visualization, Writing – review & editing. LG: Conceptualization, Supervision, Writing – review & editing. XZ: Methodology, Visualization, Writing – review & editing. YW: Investigation, Visualization, Writing – review & editing. NH: Funding acquisition, Project administration, Writing – review & editing. YY: Resources, Supervision, Writing – review & editing. XH: Conceptualization, Funding acquisition, Resources, Supervision, Validation, Writing – review & editing.

## Footnotes

^1^ https://speechbox.linguistics.northwestern.edu/
